# Supplementary material for: Inhibiting concentration quenching in Yb3+-Tm3+ upconversion nanoparticles by suppressing back energy transfer
Source: Nat Commun. 2025 May 6;16:4218. doi: 10.1038/s41467-025-59452-4 (PMC12056211; doi:10.1038/s41467-025-59452-4)
Supplement: Supplementary file 1 — Supplementary Information [file 41467_2025_59452_MOESM1_ESM.pdf]

Supplementary Information

Inhibiting Concentration Quenching in Yb^{3+}-Tm^{3+} Upconversion Nanoparticles by Suppressing Back Energy Transfer

Dingxin Huang[1,2], Feng Li[1,2], Hans Ågren[1,3] and Guanying Chen[1,2]*

[1]MIIT Key Laboratory of Critical Materials Technology for New Energy Conversion and Storage, School of Chemistry and Chemical Engineering, Harbin Institute of Technology, 150001 Harbin, People's Republic of China.

[2]Key Laboratory of Micro-systems and Micro-structures, Ministry of Education, Harbin Institute of Technology, 150001 Harbin, People's Republic of China.

[3]Department of Physics and Astronomy Uppsala University, Box 516, Uppsala SE-751 20, Sweden.

*Correspondence: chenguanying@hit.edu.cn

Supplementary Figures

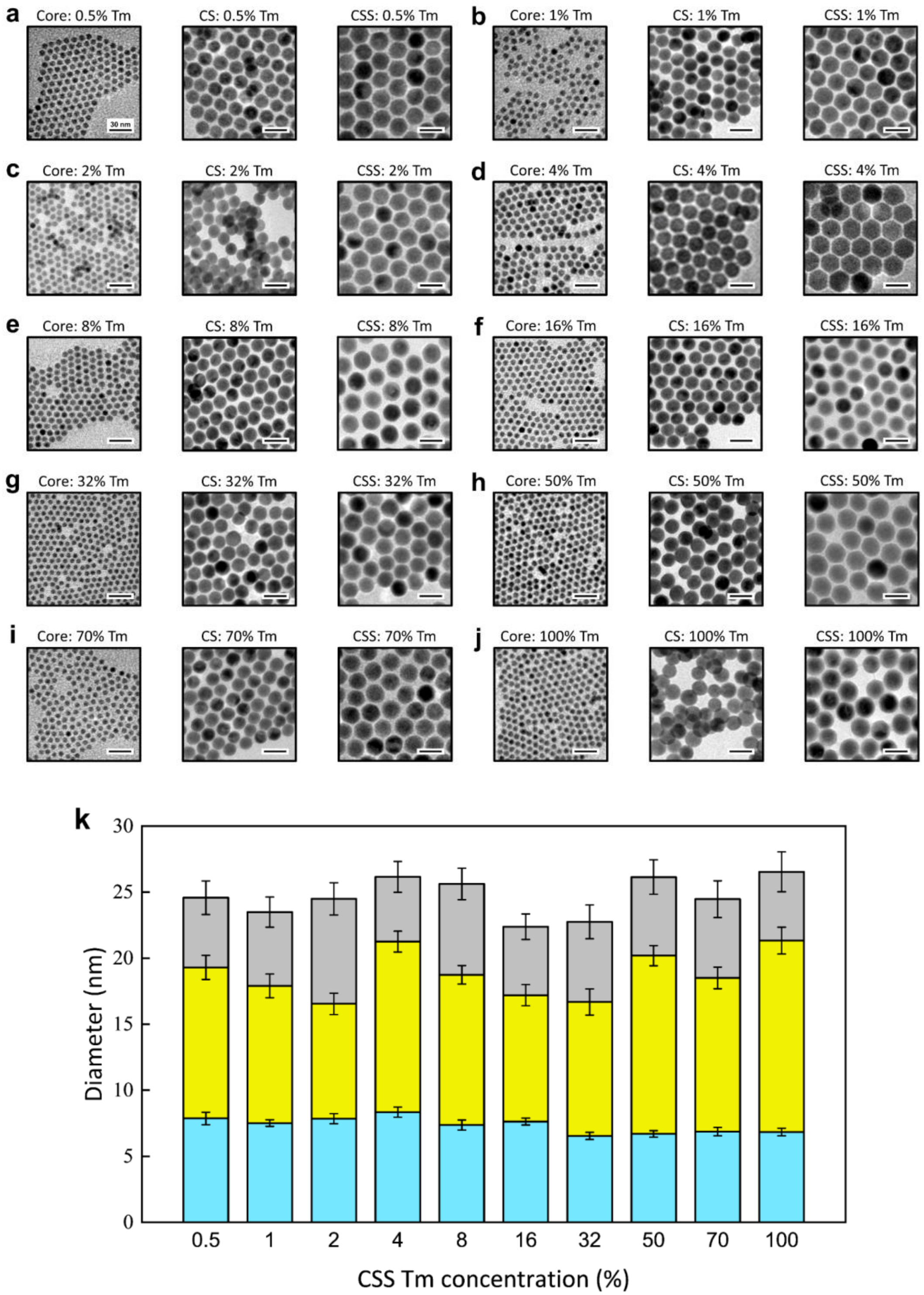

Supplementary Figure 1. Transmission electron microscopy (TEM) images of the as-synthesized core, core–shell (CS) and core–shell–shell (CSS, $NaYF_4$:x%

Tm^{3+}@$NaYbF_4$@$NaYF_4$) nanoparticles for Tm^{3+} concentration in the core of (a) 0.5%, (b) 1%, (c) 2%, (d) 4%, (e) 8%, (f) 16%, (g) 32%, (h) 50%, (i) 70% and (j) 100%. For all TEM images, scale bar is 30 nm. (k) The average diameters of the as-synthesized CSS samples doped with different Tm concentrations in (a-j). Core, CS and CSS diameter are shown as blue, yellow and gray, respectively. Error bar represents the standard deviation of the corresponding size from at least 30 nanoparticles.

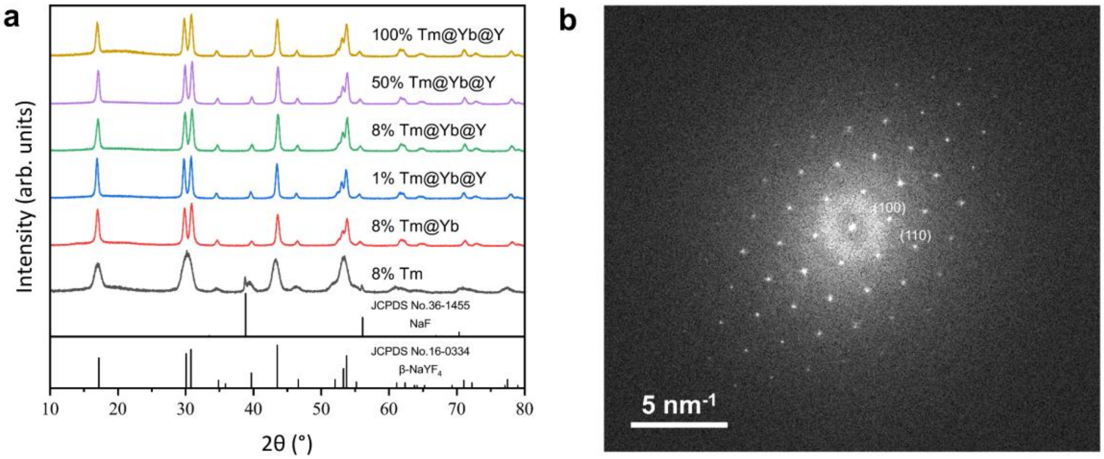

Supplementary Figure 2. Patterns of powder X-ray diffraction (XRD) and selected area electron diffraction (SAED). (a) XRD patterns for 8% Tm^{3+} core, core–shell (8% Tm^{3+} in the core) and core–shell–shell samples with varying Tm^{3+} concentration in the core. Standard patterns of hexagonal phase $NaYF_4$ (JCPDS No.16-0334) and NaF (JCPDS No.36-1455) are included for reference. NaF is the by-product during the synthesis of the core nanoparticles. (b) SAED patterns of $NaYF_4$:8% Tm@$NaYbF_4$@$NaYF_4$, matching a hexagonal $NaYF_4$ lattice.

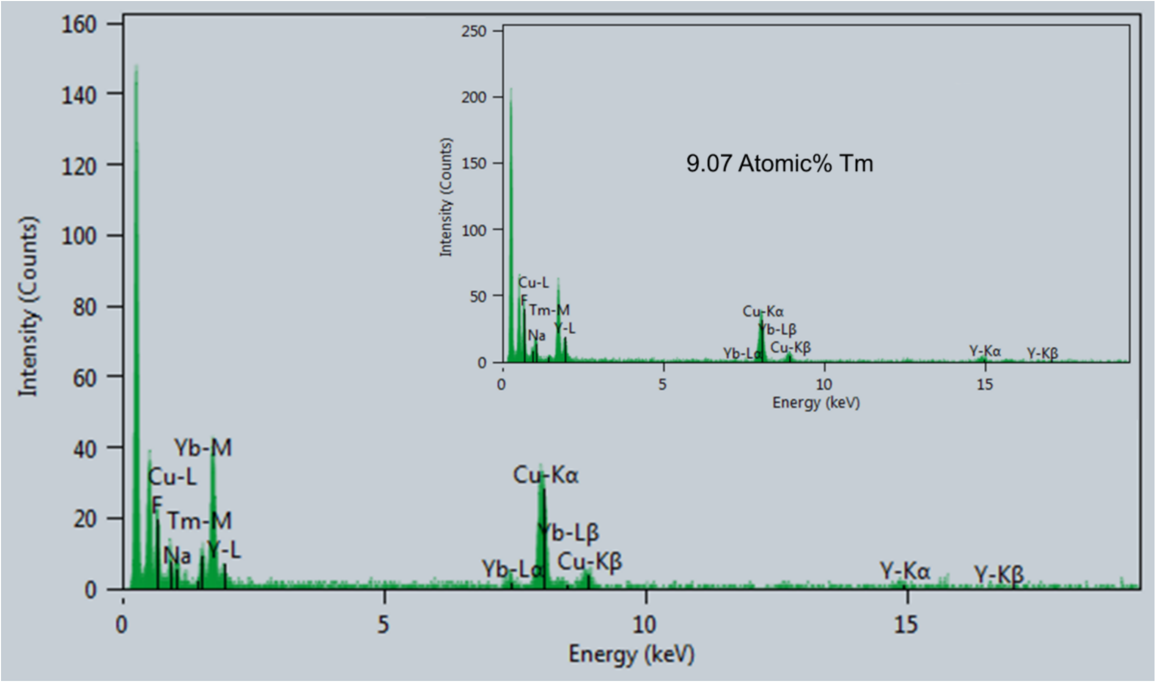

Supplementary Figure 3. Energy dispersive X-ray (EDX) spectra of $NaYF_4$:8% Tm^{3+}@$NaYbF_4$@$NaYF_4$ and $NaYF_4$:8% Tm^{3+} (inset) nanoparticles. The Tm^{3+} concentration of 9.07% determined from EDX analysis is comparable to the nominal concentration of 8 mol%.

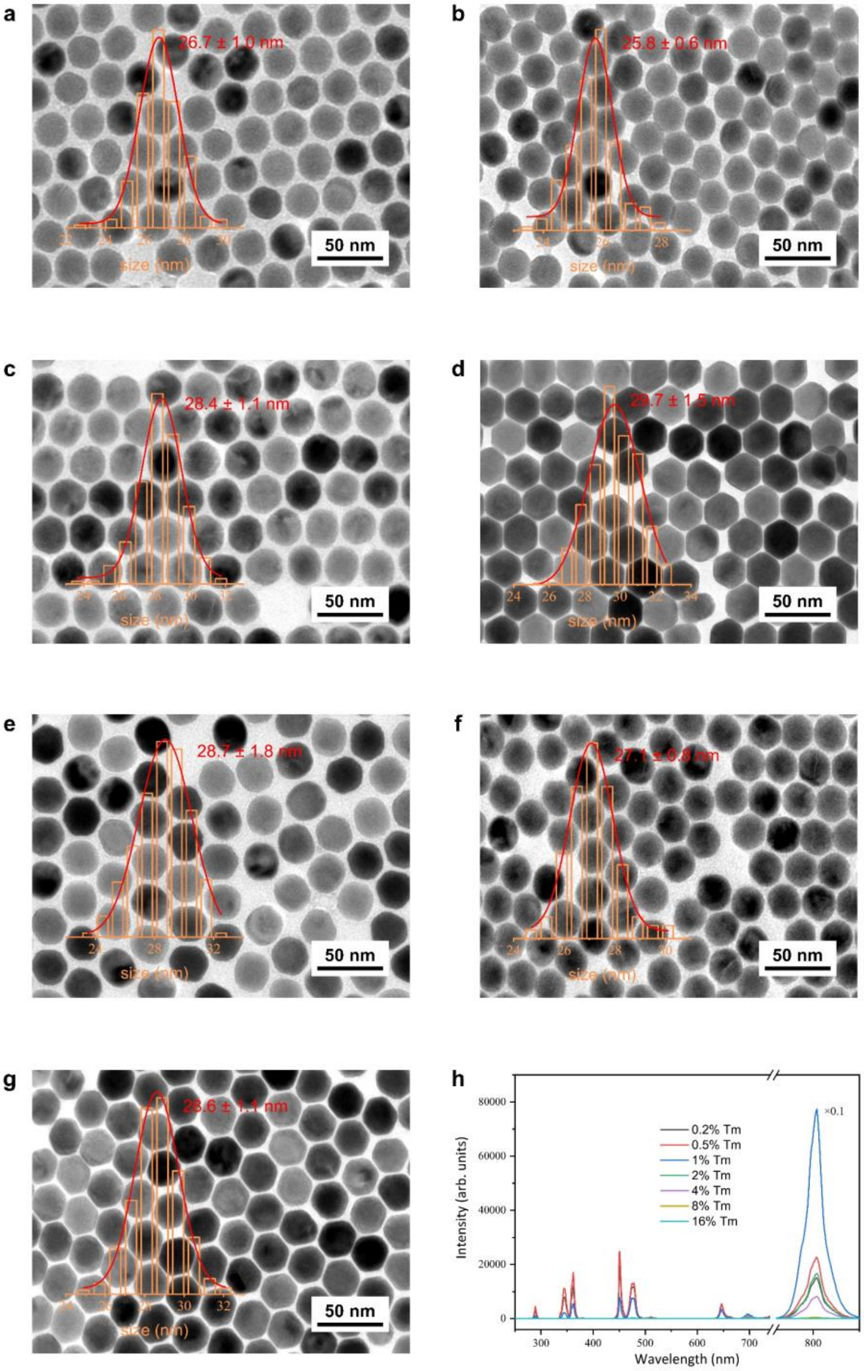

Supplementary Figure 4. TEM image of $NaYF_4$:20% Yb^{3+}, x% Tm^{3+} nanoparticles at different doping levels (a) 0.2%, (b) 0.5%, (c) 1%, (d) 2%, (e) 4%, (f) 8%, (g) 16%. The corresponding size distribution is included as the inset. All nanoparticles have an average size around 27 nm. (h) Emission spectra collected under 980-nm excitation at 99.3 W/cm^2 for upconversion nanoparticles (UCNPs) in n-hexane. The absorbance of all samples has

been normalized at 975 nm for the $^2F_{7/2} \rightarrow {}^2F_{5/2}$ transition of Yb^{3+} ions. Intensity of 800 nm emission peak multiplies by the coefficient 0.1 for better presentation of the data. Synthesis of these canonical core-only samples refers to procedures reported by Zhao et al.[1] and Zhan et al.[2] with slight modifications.

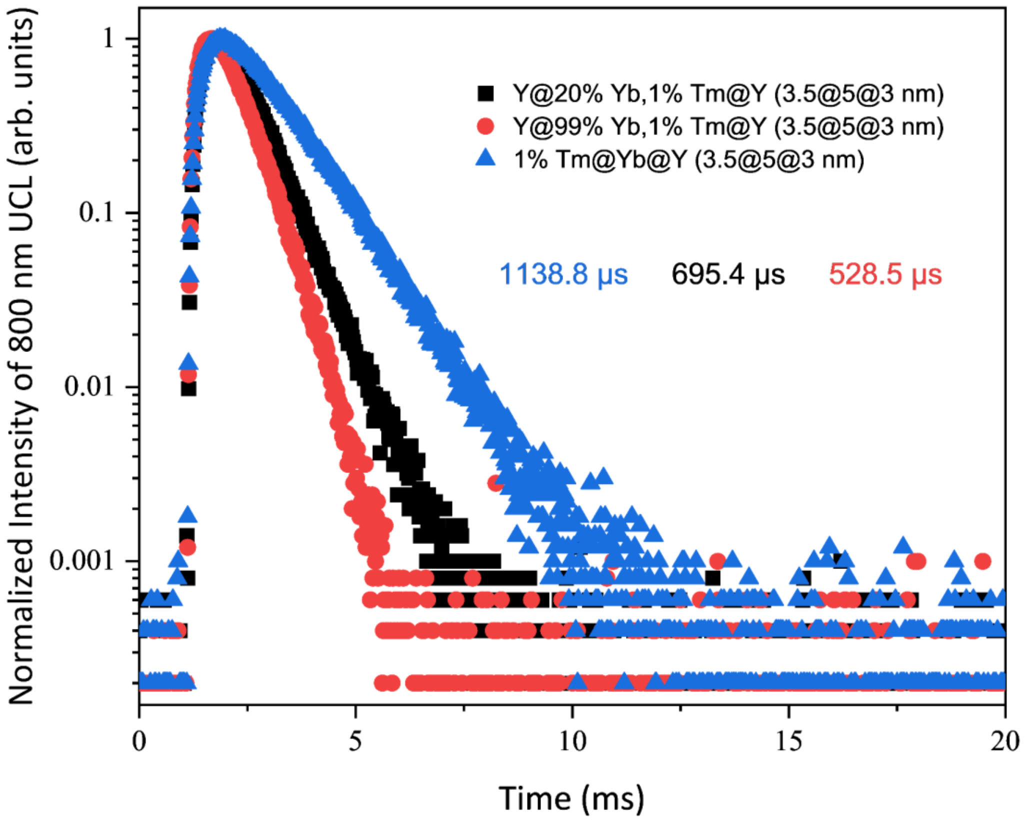

Supplementary Figure 5. Time-resolved upconversion luminescence (UCL) of the Tm^{3+} $^3H_4 \rightarrow {}^3H_6$ transition for $NaYF_4$@$NaYF_4$:20% Yb^{3+}, 1% Tm^{3+}@$NaYF_4$ (3.5@5@3 nm), $NaYF_4$@$NaYF_4$:99% Yb^{3+}, 1% Tm^{3+}@$NaYF_4$ (3.5@5@3 nm) and $NaYF_4$:1% Tm^{3+}@$NaYbF_4$@$NaYF_4$ (3.5@5@3 nm) nanoparticles measured by pulsed 980-nm excitation. The average lifetime for each sample is indicated with corresponding color. For Y@20% Yb^{3+}, 1% Tm^{3+}@Y and Y@99% Yb^{3+}, 1% Tm^{3+}@Y, the average distance between Yb^{3+} and Tm^{3+} decreases with raising of Yb^{3+} dopant concentration, which accelerates the back energy transfer (BET) from Tm^{3+} ($^3H_4$) to Yb^{3+} ($^2F_{5/2}$) inducing the decrease of Tm^{3+} lifetime. For 1% Tm^{3+}@Yb^{3+}@Y, though Yb concentration up to 100%, the average distance between Yb^{3+} and Tm^{3+} is even larger than that of Y@20% Yb^{3+}, 1% Tm^{3+}@Y as suggested by its long Tm^{3+} lifetime more than 1 ms, which reveals the role of the heterogeneous nanostructure for overcoming BET.

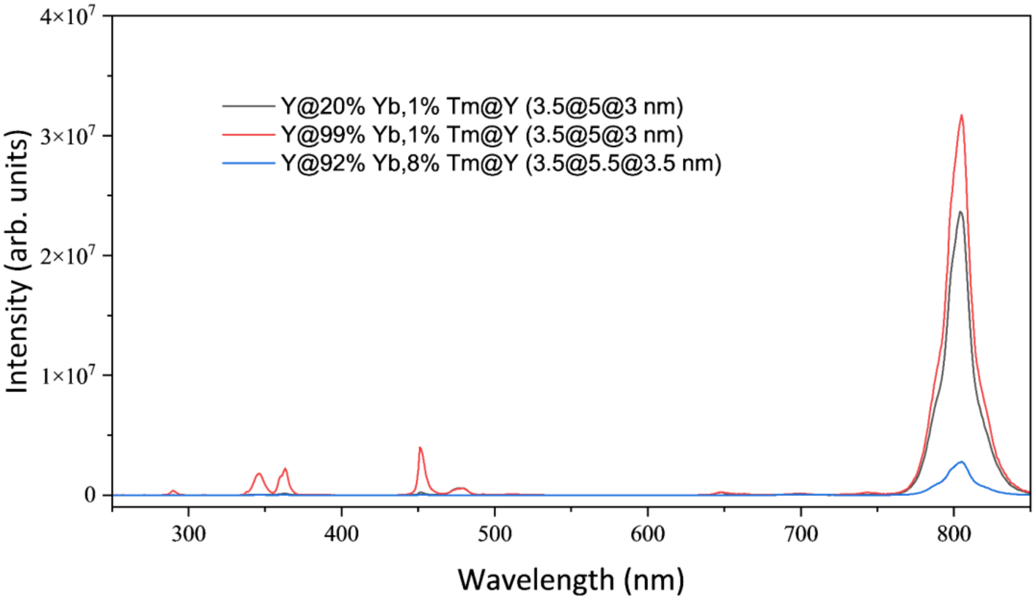

Supplementary Figure 6. Upconversion emission spectra collected under 980-nm excitation at 99.3 W/cm^2 for $NaYF_4$@$NaYF_4$:20% Yb^{3+}, 1% Tm^{3+}@$NaYF_4$ (3.5@5@3 nm), $NaYF_4$@$NaYF_4$:99% Yb^{3+}, 1% Tm^{3+}@$NaYF_4$ (3.5@5@3 nm) and $NaYF_4$@ $NaYF_4$:92% Yb^{3+}, 8% Tm^{3+}@$NaYF_4$ (3.5@5.5@3.5 nm) nanoparticles in n-hexane. The absorbance of all samples has been normalized at 975 nm for the $^2F_{7/2} \rightarrow {}^2F_{5/2}$ transition of Yb^{3+} ions. It is important to note that the concentration of nanoparticles for $NaYF_4$@$NaYF_4$: 20% Yb^{3+}, 1% Tm^{3+}@$NaYF_4$ (3.5@5@3 nm) is about 5 times as high as the other two samples in this figure, considering the absorption capacity of every nanoparticle linearly depends on its Yb^{3+} content[3]. Besides, while containing high concentration Yb^{3+} ions, $NaYF_4$@$NaYF_4$:92% Yb^{3+}, 8% Tm^{3+}@$NaYF_4$ (3.5@5.5@3.5 nm) nanoparticles exhibit relative weak upconversion luminescence due to intense back energy transfer (BET) and cross relaxation (Fig. 4c).

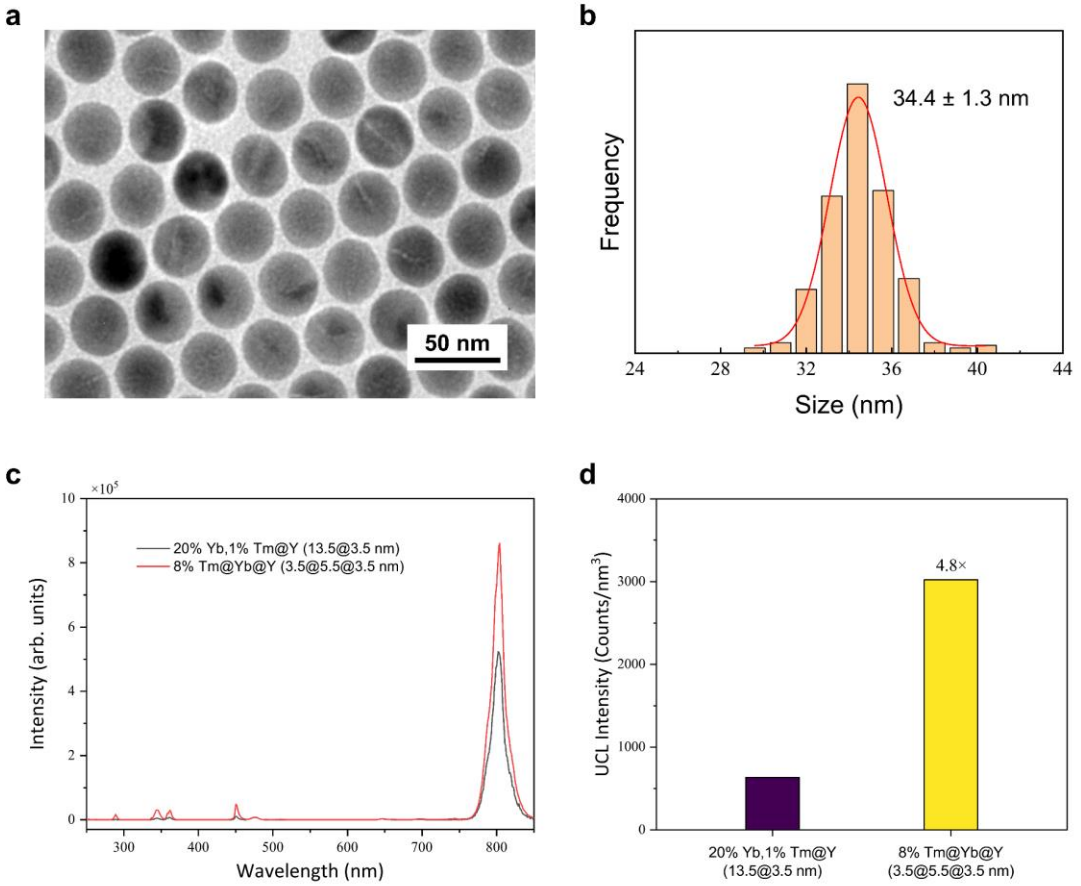

Supplementary Figure 7. TEM image (a) and size distribution (b) of state-of-the-art $NaYF_4$:20% Yb^{3+}, 1% Tm^{3+}@$NaYF_4$ (13.5@3.5 nm) core–shell nanoparticles. The size distribution is fitted by a Gaussian curve (red line). (c) Upconversion luminescence spectra for $NaYF_4$:20% Yb^{3+}, 1% Tm^{3+}@$NaYF_4$ (13.5@3.5 nm) and $NaYF_4$:8% Tm^{3+}@$NaYbF_4$ @$NaYF_4$ (3.5@5.5@3.5 nm) nanoparticles dispersed in n-hexane. The absorbance of all samples has been normalized at 975 nm for the $^2F_{7/2} \rightarrow {}^2F_{5/2}$ transition of Yb^{3+} ions. Excitation at 980 nm, under laser irradiance of 99.3 W/cm^2. (d) Compared upconversion brightness (250-850 nm) per unit volume for $NaYF_4$:20% Yb^{3+}, 1% Tm^{3+}@$NaYF_4$ (13.5@3.5 nm) and $NaYF_4$:8% Tm^{3+}@$NaYbF_4$@$NaYF_4$ (3.5@5.5@3.5 nm). The ratio of UCL brightness per unit volume for $NaYF_4$:8% Tm^{3+}@$NaYbF_4$@$NaYF_4$ to that for $NaYF_4$:20% Yb^{3+}, 1% Tm^{3+}@$NaYF_4$ is marked on the top of yellow column.

We compared the UCL brightness per unit volume (Counts/nm^3) of $NaYF_4$:8% Tm^{3+}@$NaYbF_4$@$NaYF_4$ (3.5@5.5@3.5 nm) with that of $NaYF_4$:20% Yb^{3+}, 1% Tm^{3+}@

$NaYF_4$ (13.5@3.5 nm) considering that these two kinds of nanoparticles have different overall size. The result indicated that our designed $NaYF_4$:8% Tm^{3+}@$NaYbF_4$@$NaYF_4$ (3.5@5.5@3.5 nm) nanoparticles exhibit fivefold brightness than the state-of-the-art $NaYF_4$:20% Yb^{3+}, 1% Tm^{3+}@$NaYF_4$ (13.5@3.5 nm), despite having smaller size and higher Yb^{3+} content may result in more severe surface quenching[4,5].

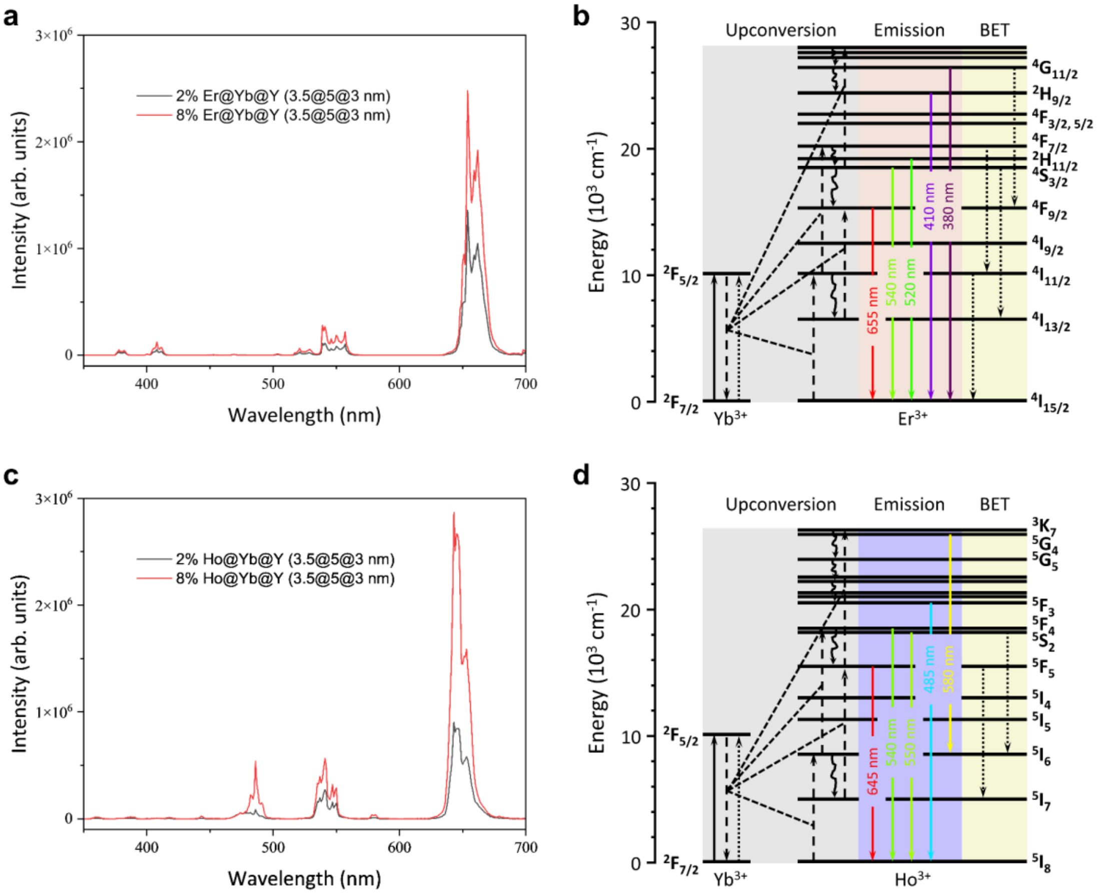

Supplementary Figure 8. Upconversion luminescence spectra collected under 980 nm laser irradiance at 99.3 W/cm^2 for (a) $NaYF_4$:2% Er^{3+}@$NaYbF_4$@$NaYF_4$ (3.5@5@3 nm) and $NaYF_4$:8% Er^{3+}@$NaYbF_4$@$NaYF_4$ (3.5@5@3 nm), (c) $NaYF_4$:2% Ho^{3+}@$NaYbF_4$@$NaYF_4$ (3.5@5@3 nm) and $NaYF_4$:8% Ho^{3+}@$NaYbF_4$@$NaYF_4$ (3.5@5@3 nm) nanoparticles in n-hexane. The absorbance of all samples has been normalized at 975 nm for the $^2F_{7/2} \rightarrow {}^2F_{5/2}$ transition of Yb^{3+} ions. Simplified energy levels of Yb^{3+}/Er^{3+} (b)[6,7] and Yb^{3+}/Ho^{3+} (d)[8-10] shows energy transfer upconversion and back energy transfer (BET) processes under 980 nm laser excitation. Solid line with an arrow, absorption or emission; curve with an arrow, multiphonon relaxation; dashed line with an arrow linked by dotted line, non-radiative energy transfer.

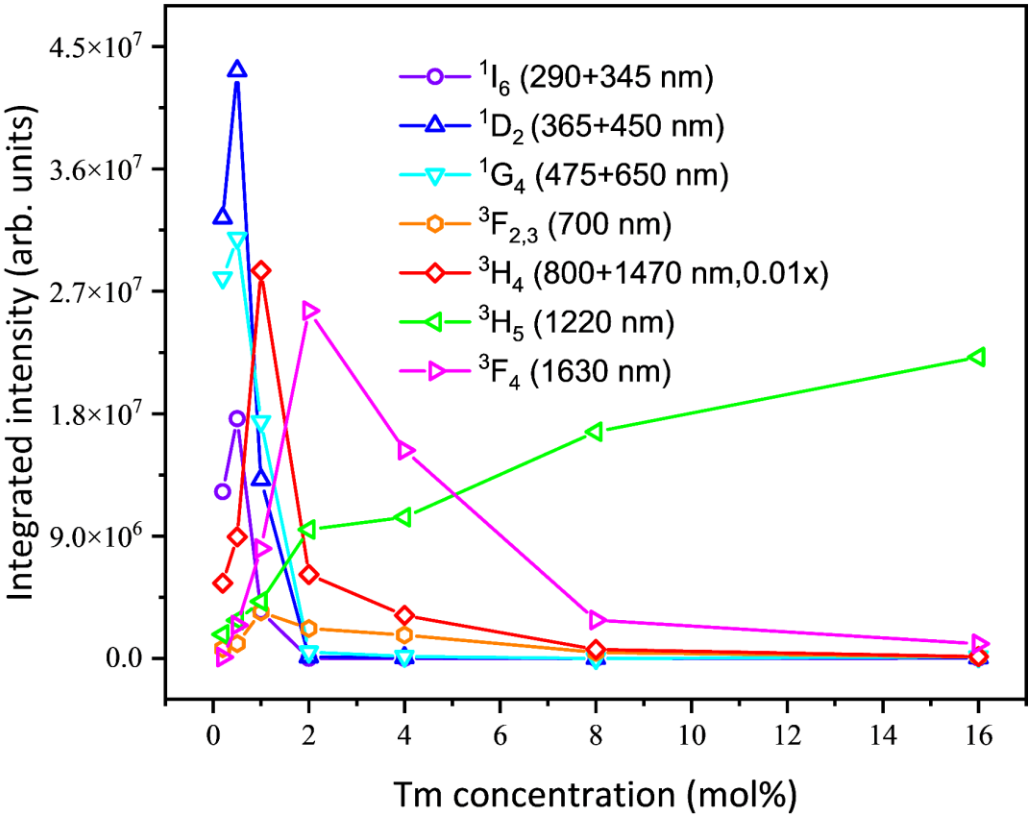

Supplementary Figure 9. The integrated emission intensity of each energy level in Tm^{3+} ions against Tm^{3+} doping concentration in $NaYF_4$:20% Yb^{3+}, x% Tm^{3+} nanoparticles (x=0.2, 0.5, 1, 2, 4, 8, 16) under 980-nm excitation at 99.3 W/cm^2. Intensity of $^3H_4$ energy level multiplies by the coefficient 0.01 for better presentation of the data.

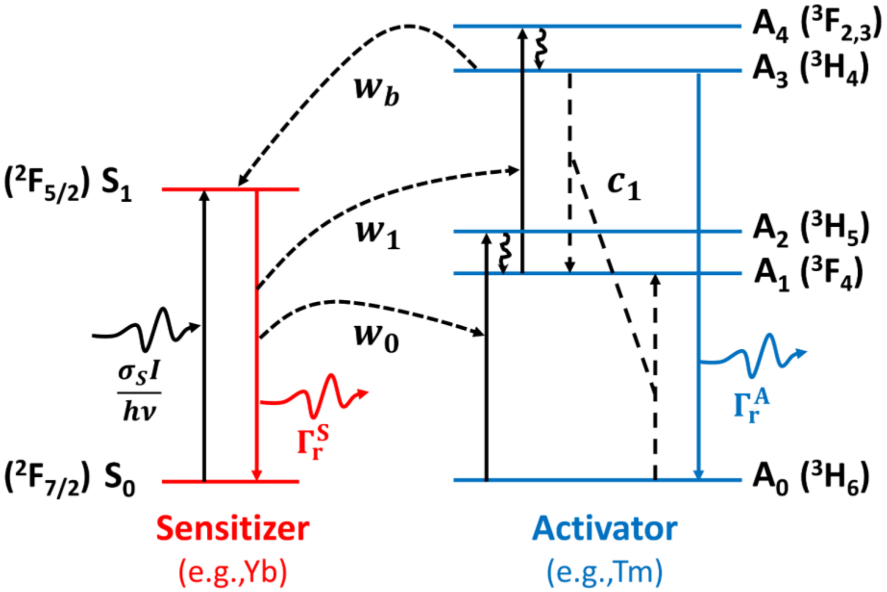

Supplementary Figure 10. The simplified energy level diagram used in modeling and simulation for concentration quenching of upconversion luminescence in Yb^{3+}-Tm^{3+} system. Given that there is relative rare population of $^1G_4$, $^1D_2$ and $^1I_6$ (Fig. 2c and Supplementary Fig. 9), the excited states above $^3F_{2,3}$ are ignored.

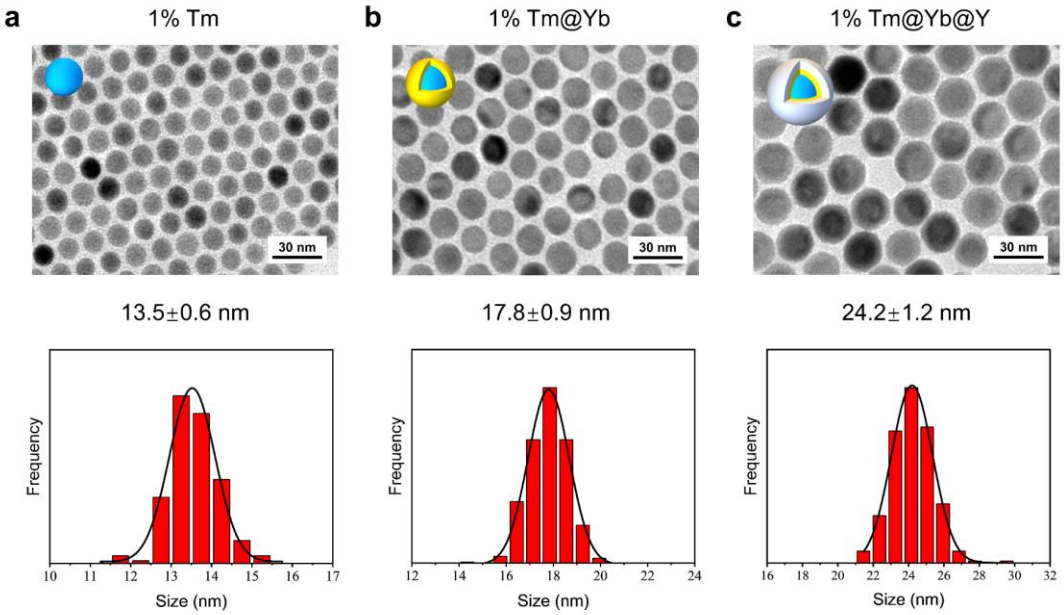

Supplementary Figure 11. TEM image and size distribution of the as-synthesized $NaYF_4$:1% Tm^{3+}@$NaYbF_4$@$NaYF_4$ (6.5@2@3 nm) core (a), core–shell (b) and core–shell–shell (c) nanoparticles. Nanoparticle size distribution is fitted by a Gaussian curve (black solid line).

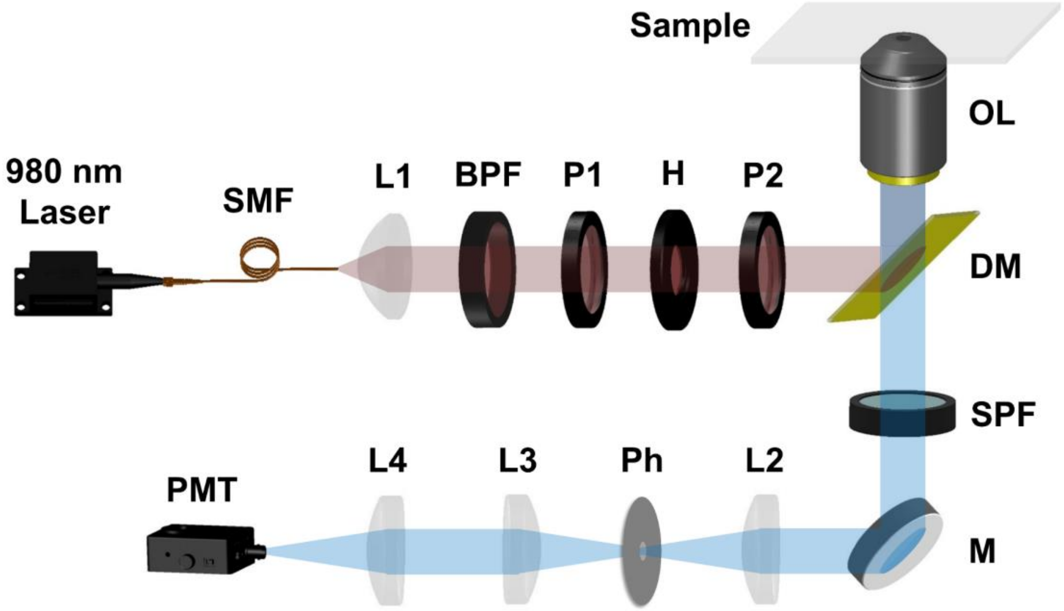

Supplementary Figure 12. Experimental setup for confocal microscopy (SMF, single-mode fiber; L1, collimation len; L2 & L3 & L4, collection lens; BPF, 980 nm band pass filter; H, half-wave plate; P1 & P2, polarized beam splitter; DM, dichroic mirror; OL, objective len; SPF, 950 nm short pass filter; M, mirror; Ph, 100 μm pinhole; PMT, photomultiplier). Partial drawing elements are sourced from "Thorlabs, Inc." under a Creative Commons Attribution License (CC BY) and the involved product part numbers are “FPL808S, P3-780Y-FC-1, ACL25416U, FBH810-10, WP25M-UB1, WPH05M-808, DMSP805R, RMS100X-PFO, NB1-K14, LA1951, APD130A”.

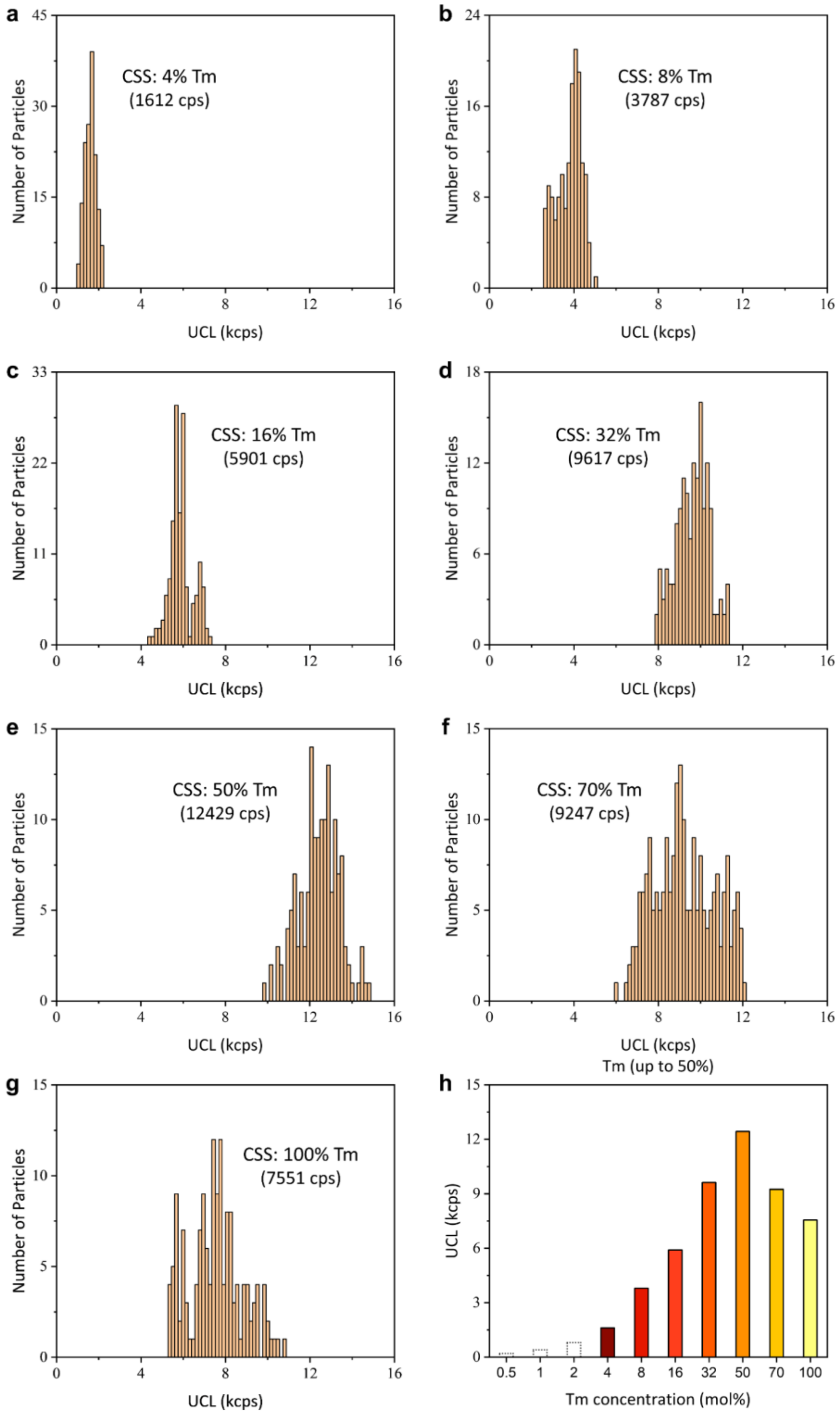

Supplementary Figure 13. Histograms of single particle brightness at 20 MW/cm^2 for core–shell–shell (CSS) UCNPs with Tm concentration of (a) 4%, (b) 8%, (c) 16%, (d) 32%, (e) 50%, (f) 70% and (g) 100% in the core. (h) Average single particle upconversion

luminescence (UCL, 400-850 nm) brightness for all 10 CSS samples under 980-nm excitation irradiance of 20 MW/cm^2; note that 0.5, 1 and 2% Tm^{3+} doped CSS samples, whose UCL brightness was denoted by the dotted box, were not bright enough to be measured.

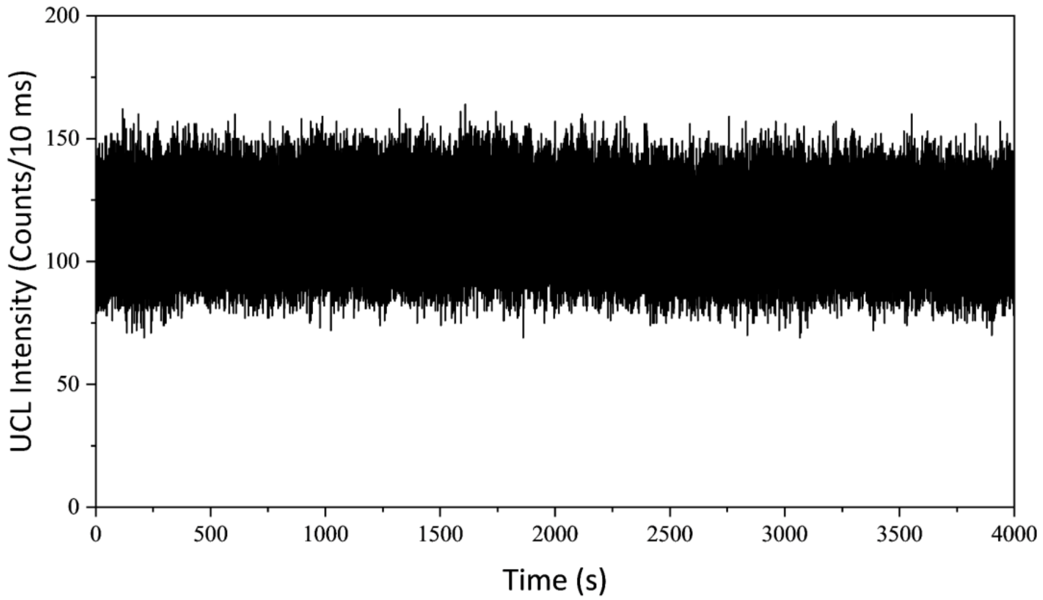

Supplementary Figure 14. The time trace of upconversion luminescence (UCL, 400-850 nm) intensity from a single nanoparticle for $NaYF_4$:50% Tm^{3+}@$NaYbF_4$@$NaYF_4$ (3.5@5@3 nm) under continuous 980-nm excitation at 20 MW/cm^2. The bin time for each data point is 10 ms.

Supplementary Table 1. The detailed Tm^{3+} doping concentration in $NaYF_4$:x% Tm^{3+} core nanoparticles determined by inductively-coupled plasma optical emission spectroscopy (ICP-OES) analysis.

$NaYF_4$:x% Tm^{3+} (nominal mol%)	ICP-OES measurement (mol%)
x = 0.5	0.3
x = 1	0.7
x = 2	1.5
x = 4	3.9
x = 8	8.1
x = 16	13.7
x = 32	27.4
x = 50	47.7
x = 70	65.6

Supplementary Table 2. The variation of integrated emission intensity of each energy level in Tm^{3+} ions with Tm^{3+} doping concentration for $NaYF_4$:x% Tm^{3+}@$NaYbF_4$@$NaYF_4$ (3.5@5@3 nm) under 980-nm excitation at 99.3 W/cm^2. Red marked numbers indicate the optimal doping concentration for each energy level. The upward sloping arrows suggest how many times the emission intensity of latter concentration compared to former. The downward sloping arrows suggest how much percentage the emission intensity of the latter concentration reduces compared to former.

Tm^{3+} (mol%)	0.5		1		2		4		8		16		32		50		70		100
$^1I_6$		6.7 ↗		12.7 ↗		1.6 ↗	4	70% ↘		97% ↘		5.8 ↗		1.7 ↗		1.2 ↗		1.9 ↗	
$^1D_2$		5.0 ↗		10.7 ↗		1.7 ↗	4	58% ↘		93% ↘		2.5 ↗		1.7 ↗		1.03 ↗		1.7 ↗	
$^1G_4$		4.3 ↗		1.8 ↗		1.02 ↗	4	74% ↘		80% ↘		1.2 ↗		1.3 ↗		1.6 ↗		1.1 ↗	
$^3F_{2,3}$		3.1 ↗		9.8 ↗		2.7 ↗		1.5 ↗	8	16% ↘		18% ↘		11% ↘		24% ↘		29% ↘	
$^3H_4$		4.4 ↗		8.9 ↗		3.5 ↗		1.04 ↗	8	49% ↘		37% ↘		18% ↘		46% ↘		21% ↘	
$^3H_5$		2.8 ↗		2.1 ↗		1.2 ↗		29% ↘		1.9 ↗		1.9 ↗		1.2 ↗		1.2 ↗		1.1 ↗	100
$^3F_4$				6.0 ↗		5.9 ↗		1.7 ↗		2.4 ↗		1.00 ↗	32	9% ↘		1.00 ↗		40% ↘	

As the emission level $^1G_4$ is populated from $^3H_4$ through energy transfer upconversion from Yb^{3+}, its optimal doping concentration would theoretically match that of $^3H_4$. However, due to the presence of a cross-relaxation process ($^1G_4 + {}^3F_4 \rightarrow {}^3F_3 + {}^3H_4$), which intensifies with increasing Tm^{3+} doping concentration. Therefore, the optimized doping concentration for $^1G_4$ is lower than that for $^3H_4$. Furthermore, the emission level $^1D_2$ is populated from $^1G_4$ by the cross-relaxation process ($^1G_4 + {}^1G_4 \rightarrow {}^1D_2 + {}^3F_3$) and $^1I_6$ is populated from $^1D_2$ by an energy transfer upconversion process from Yb^{3+}. Consequently, the optimized

doping concentration for these higher-lying emission levels ($^1G_4$, $^1D_2$, $^1I_6$) is lower than that for $^3H_4$.

Supplementary Table 3. The variation of integrated emission intensity of each energy level in Tm^{3+} ions with Tm^{3+} doping concentration for $NaYF_4$:20% Yb^{3+}, x% Tm^{3+} (13.5 nm) under 980-nm excitation at 99.3 W/cm^2. Red marked numbers indicate the optimal doping concentration for each energy level. The upward sloping arrows suggest how many times the emission intensity of latter concentration compared to former. The downward sloping arrows suggest how much percentage the emission intensity of the latter concentration reduces compared to former.

Tm^{3+} (mol%)	0.2		0.5		1		2		4		8		16
$^1I_6$		1.4 ↗	0.5	80% ↘		99% ↘		43% ↘		65% ↘		8.2 ↗	
$^1D_2$		1.3 ↗	0.5	70% ↘		99% ↘		42% ↘		78% ↘		3.7 ↗	
$^1G_4$		1.1 ↗	0.5	44% ↘		97% ↘		65% ↘		97% ↘		22.6 ↗	
$^3F_{2,3}$		1.5 ↗		3.1 ↗	1	36% ↘		21% ↘		74% ↘		75% ↘	
$^3H_4$		1.6 ↗		3.2 ↗	1	78% ↘		49% ↘		79% ↘		79% ↘	
$^3H_5$		1.6 ↗		1.5 ↗		2.3 ↗		1.1 ↗		1.6 ↗		1.3 ↗	16
$^3F_4$		24.3 ↗		3.3 ↗		3.2 ↗	2	40% ↘		82% ↘		62% ↘	

Supplementary Table 4. The rate parameters used in simulation[2,11-17] for concentration quenching of upconversion luminescence in Yb^{3+}-Tm^{3+} system ($NaYF_4$:20% Yb^{3+}, 1% Tm^{3+} core-only vs. $NaYF_4$:1% Tm^{3+}@$NaYbF_4$@$NaYF_4$ core–shell–shell (CSS) nanoparticles)

Structure	σ_S (cm^2)	w_0 (cm^{3}s^{-1})	w_1 (cm^{3}s^{-1})	w_b (cm^{3}s^{-1})	β_2 (s^{-1})	β_4 (s^{-1})
Core-only	2.0×10^{-20}	1.7×10^{-17}	3.5×10^{-16}	1.7×10^{-18}	3.4×10^{4}	1.0×10^{5}
CSS	2.0×10^{-20}	7.7×10^{-18}	1.5×10^{-16}	7.7×10^{-19}	3.4×10^{4}	1.0×10^{5}
**Structure**	**c_1 (cm^{3}s^{-1})**	**τ_{S_1} (s)**	**τ_{A_1} (s)**	**τ_{A_2} (s)**	**τ_{A_3} (s)**	**τ_{A_4} (s)**
Core-only	2.0×10^{-18}	2.0×10^{-3}	1.0×10^{-2}	5.0×10^{-3}	1.5×10^{-3}	5.0×10^{-4}
CSS	2.0×10^{-18}	2.0×10^{-3}	1.0×10^{-2}	5.0×10^{-3}	1.5×10^{-3}	5.0×10^{-4}

a) The energy transfer rate of Yb^{3+} ($^2F_{5/2}$) → Tm^{3+} ($^3H_5$), w_0, is estimated according to the corresponding value[2] of $NaYF_4$:18% Yb^{3+}, 0.5% Tm^{3+} and relationship between energy transfer rate and doping concentration[13].

b) The energy transfer rate of Yb^{3+} ($^2F_{5/2}$) → Tm^{3+} ($^3F_{2,3}$), w_1, is estimated to be 20 times as large as w_0 from de Matos, P. S. F. et al[14].

c) The back energy transfer rate of Tm^{3+} ($^3H_4$) → Yb^{3+} ($^2F_{5/2}$), w_b, is estimated to be one tenth of w_0 from de Matos, P. S. F. et al[14].

Supplementary Note

The trade-off between Yb^{3+}-Tm^{3+} energy transfer (ET) and Tm^{3+}-Yb^{3+} back energy transfer (BET) on upconversion luminescence

Separating Yb^{3+} and Tm^{3+} into different domains of the core–shell–shell nanostructure simultaneously reduces both energy transfer (ET) efficiency and back energy transfer (BET) efficiency. While the decreased ET efficiency lowers upconversion brightness, the reduced BET efficiency mitigates concentration quenching effects, thereby enhancing brightness. This trade-off in the designed core–shell–shell nanostructure ultimately results in brighter upconversion nanoparticles, as the brightness is more significantly influenced by inhibiting the concentration quenching effect.

To validate this conclusion, we synthesized a series of $NaYF_4$:x% Yb^{3+}, 4% Tm^{3+}@$NaYbF_4$@$NaYF_4$ (3.5@5@3 nm) (x=0, 5, 10, 20) as control samples (Supplementary Figure 15). Varying doping levels of Yb^{3+} ions in the core domain will simultaneously enhances both the ET efficiency from Yb^{3+} to Tm^{3+} and also the BET efficiency from Tm^{3+} to Yb^{3+}. If the ET process dominates over BET in influencing upconversion, an increase in Yb^{3+} concentration should enhance upconversion luminescence (UCL) brightness; otherwise, it would lead to a decrease. As shown in Supplementary Figure 15, increasing the Yb^{3+} doping levels in the core domain consistently reduces the UCL brightness, indicating that BET has a more pronounced effect than ET on upconversion brightness.

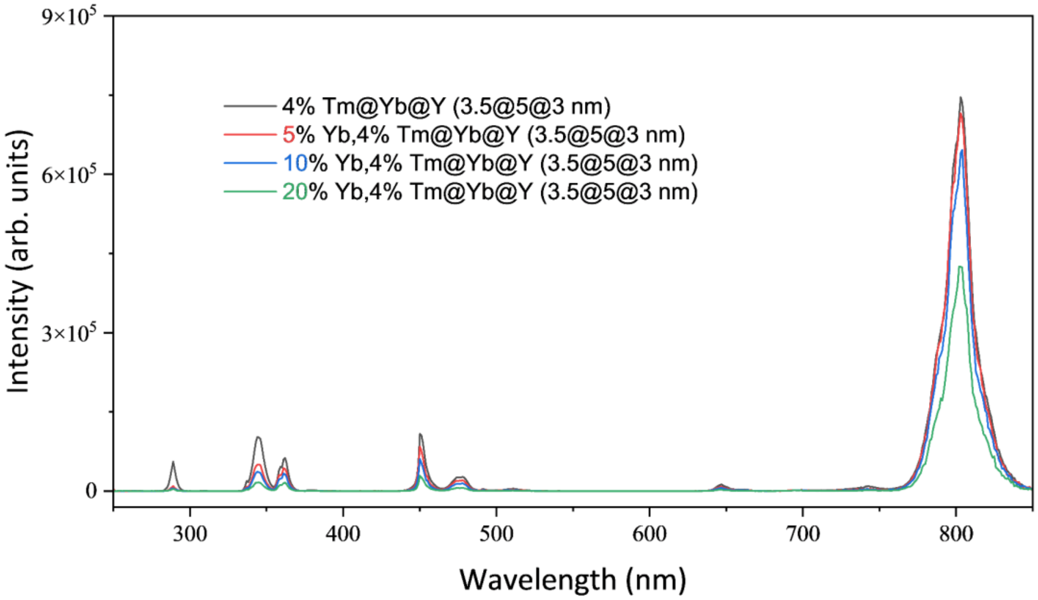

Supplementary Figure 15. Upconversion luminescence spectra collected under 980 nm laser irradiance at 99.3 W/cm^2 for $NaYF_4$:x% Yb^{3+}, 4% Tm^{3+}@$NaYbF_4$@$NaYF_4$ (3.5@5@3 nm) (x=0, 5, 10, 20) nanoparticles dispersed in n-hexane. The absorbance of all samples has been normalized at 975 nm for the $^2F_{7/2} \rightarrow {}^2F_{5/2}$ transition of Yb^{3+} ions.

To further support this conclusion, we subsequently prepared a series of $NaYF_4$:20% Yb^{3+}, x% Tm^{3+}@$NaYbF_4$@$NaYF_4$ (3.5@5@3 nm) (x=1, 2, 4, 8) control samples, in which the core domain contains additional 20 mol% Yb^{3+} dopants to enhance energy transfer from Yb^{3+} to Tm^{3+} while varying the concentrations of Tm^{3+} (Supplementary Figure 16). First, the optimal Tm^{3+} concentration was determined to be 2 mol%, in contrast to 8% for $NaYF_4$:x% Tm^{3+}@$NaYbF_4$@$NaYF_4$ samples (3.5@5@3 nm) (x=1, 2, 4, 8), respectively. This finding reconfirms the importance of spatially isolating Yb^{3+} and Tm^{3+} for inhibiting the concentration quenching effect. Second, at low Tm^{3+} dopant concentrations (x=1 and 2), $NaYF_4$:20% Yb^{3+}, x% Tm^{3+}@$NaYbF_4$@$NaYF_4$ (3.5@5@3 nm) exhibits higher UCL than $NaYF_4$:x% Tm^{3+}@$NaYbF_4$@$NaYF_4$ samples. At higher Tm^{3+} concentrations (x=4 and 8), the brightness decreases, making these samples dimmer than that of $NaYF_4$:x% Tm^{3+}@$NaYbF_4$@$NaYF_4$. This suggests that at low Tm^{3+} concentrations, ET dominates over BET in influencing UCL brightness, whereas at high Tm^{3+} concentrations, BET plays a more significant role than ET. Among all samples, the brightest one was determined to

be $NaYF_4$:8% Tm^{3+}@$NaYbF_4$@$NaYF_4$, highlighting the advantage of spatially separated Yb^{3+} and Tm^{3+} ions in the designated core–shell–shell nanostructure.

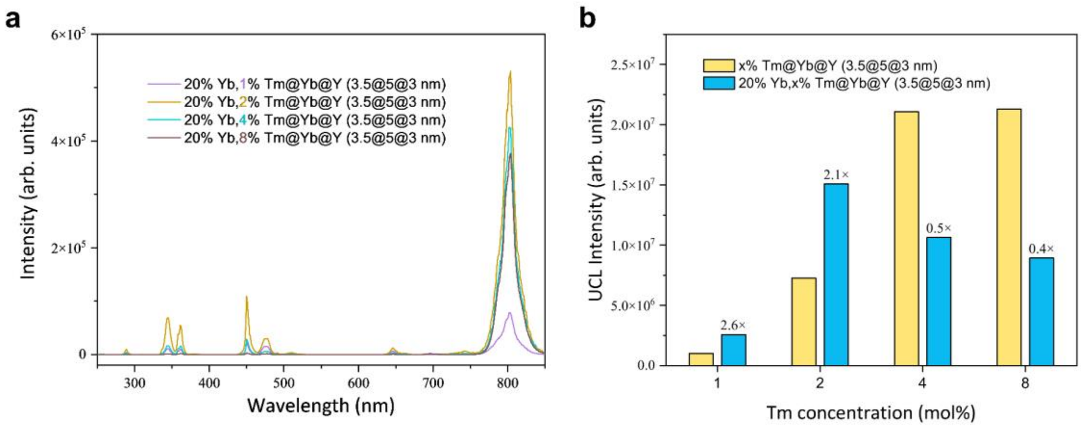

Supplementary Figure 16. Dependence of upconversion performance on Tm^{3+} concentration in the core for different Yb^{3+} spatial distributions. (a) Upconversion luminescence (UCL) spectra collected under 980 nm excitation at 99.3 W/cm^2 for $NaYF_4$:20% Yb^{3+}, x% Tm^{3+}@$NaYbF_4$@$NaYF_4$ (3.5@5@3 nm) (x=1, 2, 4, 8) nanoparticles dispersed in n-hexane. The absorbance of all samples has been normalized at 975 nm for the $^2F_{7/2} \rightarrow {}^2F_{5/2}$ transition of Yb^{3+} ions. (b) UCL intensity (250-850 nm) for $NaYF_4$:20% Yb^{3+}, x% Tm^{3+}@$NaYbF_4$@$NaYF_4$ (3.5@5@3 nm) and $NaYF_4$:x% Tm^{3+}@$NaYbF_4$@$NaYF_4$ (3.5@5@3 nm) (x=1, 2, 4, 8) under 980 nm laser irradiance of 99.3 W/cm^2. The ratio of UCL brightness for $NaYF_4$:20% Yb^{3+}, x% Tm^{3+}@$NaYbF_4$@$NaYF_4$ to that for $NaYF_4$:x% Tm^{3+}@$NaYbF_4$@$NaYF_4$ at each Tm^{3+} concentration is marked on the top of blue column for corresponding $NaYF_4$:20% Yb^{3+}, x% Tm^{3+}@$NaYbF_4$@$NaYF_4$ sample.

Supplementary Methods

Materials. Rare earth chloride hexahydrate ($YCl_3 \cdot 6H_2O$, $YbCl_3 \cdot 6H_2O$, $TmCl_3 \cdot 6H_2O$, 99.99%), rare earth oxides (Y_2O_3, Yb_2O_3, Tm_2O_3, 99.99%), trifluoroacetic acid (TFA, 99%), sodium trifluoroacetate (CF_3COONa, 97%), sodium oleate (>97%), sodium hydroxide (NaOH, 99.9%) were all purchased from Aladdin. Oleic acid (OA, technical grade, 90%), 1-octadecene (ODE, technical grade, 90%) and oleylamine (OM, technical grade, 70%) were purchased from Sigma-Aldrich. Other chemical reagents, such as ammonium fluoride, methanol, absolute ethyl alcohol and n-hexane, were acquired from Sinopharm Chemical Reagent Co., Ltd., Beijing, China. All materials were used as received without further purification.

Preparation of $Re(CF_3COO)_3$ (Re=Y, Yb, Tm). $Re(CF_3COO)_3$ (Re=Y^{3+}, Yb^{3+}, Tm^{3+}) was prepared using the following procedure adapted from our previous publication[18]. Firstly, 2 mmol corresponding rare earth oxides were dissolved in 20 ml TFA solution of 50% (v/v) under heating at 90℃ in a three-neck flask, yielding a transparent solution. Subsequently, the solution was evaporated to dryness under argon gas protection. The final $Re(CF_3COO)_3$ (Re=Y, Yb, Tm) of white powder was collected for further use.

Preparation of Yb(Y)-OA precursor for the energy migration (outmost) shell. The Yb(Y)-OA precursor was prepared following a literature method with adaptations[19]. Typically, 4 mmol $Yb(CF_3COO)_3$ was added into a 100 mL three-neck flask containing 4 mL OA, 6 mL ODE and 1 mL OM. Then, the mixture solution was heated to 120℃ and maintained at this temperature for 45 min under argon gas protection, yielding a clear and slightly yellow solution. After naturally cooling down to room temperature, the viscous precursor liquid was stored in a glass vial for further use.

Preparation of (100-x)% Yb, x% Tm-OA precursor. The procedure of preparing (100-x)% Yb, x% Tm-OA precursor is identical to that for Yb-OA precursor, except for using a mixture of 4 mmol $Re(CF_3COO)_3$ with the corresponding molar ratio of (100-x)% $Yb(CF_3COO)_3$, x% $Tm(CF_3COO)_3$.

Preparation of Na-OA shell precursors for all shells. 4 mmol CF_3COONa was added into a 10 mL glass vial containing 4.5 mL OA and 4.5 mL ODE. The mixture was ultra-sounded until a complete dissolution of the solid CF_3COONa, yielding a clear solution. Then, the vial was stored at room temperature for further use.

The final precursor for shelling growth was obtained by mixing aliquots of each Re-OA (Re=Y^{3+}, Yb^{3+}, Yb^{3+}/Tm^{3+}) and Na-OA precursor (Re:Na=1:1) into a 20 mL vial under ultrasound for half an hour, yielding molar concentration of both rare-earth oleate and Na-OA to be 0.2 M.

General procedure for synthesis of the core nanoparticles. The β-$NaYF_4$:x% Tm^{3+} core nanoparticles with a small size of 7 nm were prepared following our previously reported method with adaptations[20].

First, Re-OA precursor was prepared using the following procedure. 1 mmol $ReCl_3 \cdot 6H_2O$ (Re = Y^{3+}, Tm^{3+} and Y^{3+}:Tm^{3+} = (100-x)%:x%) and 3 mmol sodium oleate were dissolved in a mixed solution containing 3 mL deionized water, 3.5 mL absolute ethyl alcohol and 7 mL n-hexane. The resulting solution was heated to 60℃ and kept at this temperature for 12 h. Then, a transparent organic phase containing Re-OA was obtained after washing the final complex three times with deionized water in a separatory funnel.

Second, the obtained Re-OA precursor was mixed with 8 mmol sodium oleate, 5.2 mL OA, 5.1 mL OM and 9 mL ODE. The temperature was then raised up to 100℃ under argon flow with vigorous stirring to remove water and hexane. After aging for 60 min, 8 mmol solid ammonium fluoride was added into the above solution and vigorously stirred for 30 min at 100℃. Subsequently, the reaction mixture was heated to 300℃ at the rate of 10 K/min for half an hour. After naturally cooling down to room temperature, the reaction solution was supplemented with an equal volume of ethanol, and the resulting nanoparticles was collected via centrifugation at 8000 rpm for 5 min. The particles were then purified by redispersing the precipitate in 3 mL of hexane, followed by washing with ethanol several times. The final product was dispersed in 10 mL n-hexane for further use.

General procedure for synthesis of the core–shell nanoparticles. The β-$NaYF_4$:x% Tm^{3+}@$NaYbF_4$ nanoparticles with 5 nm shell thickness were prepared following a

literature hot-injection method with adaptations[19]. Typically, 5 mL (0.5 mmol) hexane dispersed β-$NaYF_4$:x% Tm^{3+} core nanoparticles were first mixed with 6 mL OA and 15 mL ODE in a 250 mL three-neck flask, the mixture was heated to 120℃ for 30 min, and then to 300℃ at the rate of 10 K/min under argon gas protection. Subsequently, an aliquot of energy migration shell precursor (1 mL) that contains 0.2 mmol Yb-OA and 0.2 mmol Na-OA was consecutively injected into the solution per an interval of 10 min. Totally 20 mL shell precursor resulted in 5 nm $NaYbF_4$ shell thickness. After completion of shell growth, the reaction was allowed to cool down to room temperature by removing the heating mantle. The resultant β-$NaYF_4$:x% Tm^{3+}@$NaYbF_4$ nanoparticles were collected using the identical procedure as that for the parent β-$NaYF_4$:x% Tm^{3+} core nanoparticles, and then dispersed in 10 mL hexane for further use.

General procedure for synthesis of the core–shell–shell nanoparticles. The procedure of synthesizing β-$NaYF_4$:x% Tm^{3+}@$NaYbF_4$@$NaYF_4$ nanoparticles, with 3 nm $NaYF_4$ shell thickness, is identical to that for the preparation of β-$NaYF_4$:x% Tm^{3+}@$NaYbF_4$ nanoparticles. The difference lies in the use of β-$NaYF_4$:x% Tm^{3+}@$NaYbF_4$ to replace the core β-$NaYF_4$:x% Tm^{3+}, and the use of $NaYF_4$ shell precursor to substitute the $NaYbF_4$ shell precursor. A total volume of 8 mL precursor is injected for the growth of $NaYF_4$ shell (3nm).

Synthesis of β-$NaYF_4$:20% Yb^{3+}, x% Tm^{3+} nanoparticles. The β-$NaYF_4$:20% Yb^{3+}, x% Tm^{3+} nanoparticles with a size of 27 nm were prepared using the coprecipitation protocol. A typical procedure is as follows: 0.01*(80-x) mmol $YCl_3\cdot 6H_2O$, 0.2 mmol $YbCl_3\cdot 6H_2O$ and 0.01*x mmol $TmCl_3\cdot 6H_2O$ were added into a 250 mL three-neck round-bottom flask containing 6 mL OA and 15 mL ODE. The mixture was heated to 160°C at the rate of 10 K/min and maintained at 160°C for 40 min under argon gas protection with magnetic stirring, yielding a clear yellowish solution. After naturally cooling down to room temperature, 10 mL methanol solution containing 2.5 mmol NaOH and 4 mmol NH_4F was quickly injected into the flask. The mixture was heated to 50°C at the rate of 10 K/min and maintained at 50°C for 30 min. Then, the mixture was heated to 110°C at the rate of 10 K/min and maintained at 110°C for 30 min. After degassing, the mixture was heated to

300°C at the rate of 10 K/min and maintained at 300°C for 1 h. After naturally cooling down to room temperature, the final reaction solution was transferred to double 50 mL centrifugal tubes with an equal volume. The resulting β-$NaYF_4$:20% Yb^{3+}, x% Tm^{3+} nanoparticles were precipitated by addition of ethanol with same volume as an aliquot of reaction solution, collected by centrifugation at 8000 rpm for 5 min and dispersed by dissolution in hexane. Subsequently, the nanoparticles were washed twice more by adding ethanol, centrifuging and dissolving in hexane. The purified nanoparticles were stored in 10 mL hexane for further use.

Supplementary References

1. Zhao, J. et al. Single-nanocrystal sensitivity achieved by enhanced upconversion luminescence. *Nat. Nanotechnol.* **8**, 729-734 (2013).
2. Zhan, Q. et al. Achieving high-efficiency emission depletion nanoscopy by employing cross relaxation in upconversion nanoparticles. *Nat. Commun.* **8**, 1058 (2017).
3. Chen, G., Ohulchanskyy, T. Y., Kumar, R., Ågren, H. & Prasad, P. N. Ultrasmall monodisperse $NaYF_4$:Yb^{3+}/Tm^{3+} nanocrystals with enhanced near-infrared to near-infrared upconversion photoluminescence. *ACS Nano* **4**, 3163-3168 (2010).
4. Gargas, D. J. et al. Engineering Bright Sub-10-nm Upconverting Nanocrystals for Single-Molecule Imaging. *Nat. Nanotechnol.* **9**, 300-305 (2014).
5. Ma, C. et al. Optimal Sensitizer Concentration in Single Upconversion Nanocrystals. *Nano Lett.* **17**, 2858-2864 (2017).
6. Frenzel, F. et al. Multiband emission from single β-$NaYF_4$(Yb,Er) nanoparticles at high excitation power densities and comparison to ensemble studies. *Nano Res.* **14**, 4107-4115 (2021).
7. Zhou, B. et al. Enhancing multiphoton upconversion through interfacial energy transfer in multilayered nanoparticles. *Nat. Commun.* **11**, 1174 (2020).
8. Pilch, A. et al. The concentration dependent up-conversion luminescence of Ho^{3+} and Yb^{3+} co-doped β-$NaYF_4$. *J. Lumin.* **182**, 114-122 (2017).
9. Li, M. et al. Impact of high sensitizer doping on the transient multiband upconversion luminescence in β-$NaYF_4$:Yb/Ho microcrystals. *Opt. Mater.* **156**, 115993 (2024).
10. Kuang, Y. et al. Fine-Tuning Ho-Based Red-Upconversion Luminescence by Altering $NaHoF_4$ Core Size and $NaYbF_4$ Shell Thickness. *Chem. Mater.* **31**, 7898-7909 (2019).
11. Dawson, J. W. et al. Analysis of the scalability of diffraction-limited fiber lasers and amplifiers to high average power. *Opt. Express* **16**, 13240-13266 (2008).
12. Schimpf, D. N., Limpert, J. & Tünnermann, A. Optimization of high performance ultrafast fiber laser systems to > 10 GW peak power. *J. Opt. Soc. Am. B* **27**, 2051-2060 (2010).

13. Lee, C. et al. Giant nonlinear optical responses from photon-avalanching nanoparticles. *Nature* **589**, 230-235 (2021).

14. de Matos, P. S. F. et al. Energy-transfer processes in high power Yb:Tm:YLF lasers emitting at 2.3 μm. *AIP Conf. Proc.* **992**, 386-391 (2008).

15. Labbaci, K. & Diaf, M. Crystal growth and spectroscopic investigations of Tm^{3+} ions doped 5NaF-9YF_3 fluoride single crystals. *Phys. Scr.* **75**, 327 (2007).

16. Loiko, P. et al. Emission properties of Tm^{3+}-doped CaF_2, KY_3F_{10}, $LiYF_4$, $LiLuF_4$ and BaY_2F_8 crystals at 1.5 μm and 2.3 μm. *J. Lumin.* **225**, 117279 (2020).

17. Diaf, M. et al. Synthesis and spectroscopic studies of Tm^{3+}-doped KY_3F_{10} single crystals. *Can. J. Phys.* **77**, 693-697 (2000).

18. Tan, M. et al. Rare-earth-doped fluoride nanoparticles with engineered long luminescence lifetime for time-gated in vivo optical imaging in the second biological window. *Nanoscale* **10**, 17771-17780 (2018).

19. Fischer, S., Bronstein, N. D., Swabeck, J. K., Chan, E. M. & Alivisatos, A. P. Precise tuning of surface quenching for luminescence enhancement in core–shell lanthanide-doped nanocrystals. *Nano Lett.* **16**, 7241-7247 (2016).

20. Li, H., Xu, L. & Chen, G. Controlled synthesis of monodisperse hexagonal NaYF(4):Yb/Er nanocrystals with ultrasmall size and enhanced upconversion luminescence. *Molecules* **22**, 2113 (2017).
